# Immunoglobulin A Nephropathy Presenting After Streptococcal Pharyngitis: A Case Report

**DOI:** 10.7759/cureus.46531

**Published:** 2023-10-05

**Authors:** Stedrea Hutchinson, Nicholas Pereira

**Affiliations:** 1 College of Medicine, Saint James School of Medicine St. Vincent, Arnos Vale, VCT; 2 Pediatrics, South Texas Health System Children’s, Edinburg, USA

**Keywords:** iga glomerulonephritis, nephropathy, iga nephropathy, igan, immunoglobulin a nephropathy

## Abstract

Immunoglobulin A (IgA) nephropathy (IgAN) is a condition characterized by IgA glomerular deposits with local cellular proliferation on renal biopsy. IgAN is the most common glomerular disease and the most common cause of renal failure worldwide. IgAN’s pathogenesis is hypothesized as a four-hit process causing improper IgA production. Four-hit processes result in an increased level of circulating galactose deficient IgA1 (Gd-IgA1). In response, Gd-IgA1 is perceived as an autoantigen by other antibodies leading to renal tissue injury. This report outlines a case of a 15-year-old Hispanic male who presented to the Emergency Department with complaints of fever and hematuria, a week after testing positive for streptococcal pharyngitis. This case summarizes the presentation and management of IgAN.

## Introduction

Immunoglobulin A (IgA) nephropathy (IgAN) is the most common glomerular disease worldwide, as well as the cause of renal failure [1]. It was noted by Jean Berger that a patient population found to have IgAN had macroscopic hematuria following episodes of pharyngitis [1-4]. IgAN is an autoimmune process characterized by renal biopsy by IgA glomerular deposits with local cellular proliferation. In addition, macroscopic hematuria and proteinuria are a consequence of the autoimmune process [1-4]. This article describes a case of a 15-year-old Hispanic male who presented to the Emergency Department (ED) with complaints of a fever starting three days prior and gross hematuria. The patient was diagnosed with streptococcal pharyngitis a week before the presentation to the ED. A diagnosis of IgAN was made via percutaneous kidney biopsy under real-time ultrasound guidance. Results of the pathology investigation were consistent with IgAN; mild mesangial hypercellularity due to diffuse 2+ granular mesangial staining was noted and IgA was visualized.

## Case presentation

A 15-year-old Hispanic male presented to the ED with complaints of a three-day fever (Table 1). In the ED, the patient’s temperature measured 102 degrees Fahrenheit. The patient confirmed experiencing associating symptoms of sore throat, headache, and most notably, gross hematuria that commenced the night prior. Also, the patient was diagnosed with streptococcal pharyngitis a week prior at their primary care institution. In addition, the patient’s medical history was notable for asthma.

**Table 1:** Laboratory Panel completed in Emergency Department

Test	Result	Reference Range
Hemoglobin	13.80 gm/dL	10.5-13.5 gm/dL
Hematocrit	42.00%	33-39%
Red Blood Cells	5.19 x10^6^/mL	3.7-5.3 x10^6^/mL
White Blood Cells	16.60 x10^3^/mL	6.0-17.5 x10^3^/mL
Platelets	138 x10^3^/mcL	150-375 10^3^/mcL
Sodium	134 mmol/L	132-140 mmol/L
Potassium	3.5 mmol/L	3.5-6.3 mmol/L
Chloride	99 mmol/L	97-106 mmol/L
Urine Color	Light-Orange	-
Urine Clarity	Turid	-
Urine Specific Gravity	1.026	-
Urine pH	6.0 pH units	-
Urine Leukocyte Esterase	1+	-
Urine Nitrite	Negative	-
Urine Protein	3+ mg/dL	-
Urine Glucose	Negative mg/dL	-
Urine Ketones	1+ mg/dL	-
Urine Urobilinogen	Negative mg/dL	-
Urine Bilirubin	Negative mg/dL	-
Urine Blood	3+ mg/dL	-
Urine WBC	30-49 /HPF	-
Urine RBC	>150 /HPF	-
Urine Bacteria	1+ /HPF	-
Urine Squamous Epithelial	None /HPF	-
Urine Mucous	Trace /HPF	-

Nephrology was consulted and ordered a renal ultrasound, which was unremarkable, as well as additional labs (Table 2).

**Table 2:** Additional Laboratory Panel Ordered by Nephrology Consult

Test	Result	Reference Range
Antistreptolysin O Screen	46 units/mL	0-250 units/mL
C3	109.43 mg/dL	88-201 mg/dL
C4	28.08 mg/dL	15-45 mg/dL

Based on additional laboratory results, Nephrology concluded that the patient was not experiencing post-streptococcal glomerulonephritis. Anti-streptolysin O (ASO) demonstrated a normal range as well as C3 and C4 also being in a normal range, therefore making post-streptococcal glomerulonephritis unlikely. Nephrology then considered IgAN due to the patient's clinical presentation. A percutaneous kidney biopsy under real-time ultrasound guidance was completed with the right native kidney being visualized with an ultrasound probe, with three cores of kidney tissues obtained from the lower pole of the right kidney. The specimen was then sent to a pathology laboratory for analysis. 

Direct immunofluorescence showed mild mesangial hypercellularity due to diffuse 2+ granular mesangial staining noted with IgA with accompanying 2-3+ staining with C3 and lambda and trace 1+ staining with kappa. There were a few small intratubular cast stains for IgA and equally for kappa and lambda. The remaining stains for IgG, IgM, C1q, albumin, and fibrinogen were all negative. In conclusion, there was a final diagnosis of IgAN.

## Discussion

IgAN is the most common glomerular disease worldwide, with an incidence that is highest in East Asia and lowest in North America and Europe [1-4]. Also, IgAN is the most common cause of renal failure [1-4]. IgAN tends to present after an infectious etiology [1]. It was noted by Jean Berger that a patient population found to have IgA nephropathy had macroscopic hematuria following episodes of pharyngitis [1]. Various infections including malaria, chlamydia, and Lyme disease have been implicated in the pathogenesis of IgAN. Among the viral infections, Epstein-Barr virus, respiratory syncytial virus, adenovirus, and severe acute respiratory syndrome coronavirus 2 (SARS‑CoV‑2) infection are worth mentioning [5-6].

The symptoms of IgAN range from microscopic to macroscopic hematuria, and nephritic and nephrotic syndromes. Macroscopic hematuria is more frequently present in children with IgAN [1]. IgAN has been noted to progress one’s risk of developing end-stage renal disease (ESRD). It is estimated that five years after an IgAN diagnosis, one’s risk of developing ESRD is 5-15%. However, 20 years after an IgAN diagnosis, one’s risk of developing ESRD is as high as 10-50% [1]. 

IgAN’s pathogenesis is hypothesized as a four-hit process causing improper IgA production [1]. The pathogenesis of IgAN concerns the IgA1 hinge region at its carbohydrate side chains. Overall, IgA is the most prominent immunoglobulin and has two subclasses, IgA1 and IgA2. The two subclasses are differentiated by the length of their heavy chain hinge regions, IgA1 having the longer hinge region [5]. 

IgA1 hinge region consists of O-linked glycans composed of N-acetylgalactosamine or sialylated N-acetylgalactosamine [5]. The N-acetylgalactosamine or sialylated N-acetylgalactosamine portions can consist of galactose or be galactose deficient. In the case of IgAN, it is exclusively caused by mesangial IgA1 that is galactose deficient, Gd-IgA1 [5]. 

The four hit processes consist of increased levels of circulating Gd-IgA1 (hit 1). In response, Gd-IgA1 is perceived as an autoantigen by other antibodies, mostly IgG (hit 2). Gd-IgA1 and antibodies form immune complexes (hit 3). Immune complexes then deposit in the glomeruli and cause kidney damage (hit 4) [5]. 

The presence of immune complexes causes mesangial cell proliferation, and activation of pro-inflammatory and pro-fibrotic cytokines that lead to further injury to podocytes and tubular epithelial cells of the kidney [5]. Consequently, there is an increase in glomerular permeability, causing proteinuria and hematuria. Increased duration and injury may also manifest with hypertension and decreased renal function [5].

Specifically, clinical studies have shown that variants of the alternative and lectin pathways of complement systems have a link between IgA deposition and glomerular inflammation [3]. However, the classical pathway can be detected by C1q immunostaining, which is vastly absent in a majority of IgAN kidney biopsies, suggesting that the classical pathway does not contribute to IgAN pathogenesis [3]. Also, IgAN is characterized by the presence of dominant or co-dominant mesangial IgA deposits, often accompanied by complement component 3 (C3) and immunoglobulin G (IgG) [2]. However, IgG deposits are not essential to the definitive diagnosis of IgAN but their presence indicates poor outcomes [2]. In the present case, IgG was negative. 

With regard to treatment for IgAN, patients with insignificant disease activity, meaning normal blood pressure, normal estimated glomerular filtration rate (eGFR), no microscopic hematuria, and urine protein-to-creatinine ratio persistently less than 0.2g/g require no treatment [5]. However, those who persistently express proteinuria benefit from treatments that suppress the renin-angiotensin system (RAS), such as an angiotensin-converting enzyme (ACE) inhibitor or an angiotensin II receptor blocker [5]. Such treatments will address blood pressure control and proteinuria reduction. If conservative medication does not work, various immunosuppressive treatments such as corticosteroids have been supported in reducing proteinuria [5].

## Conclusions

This article summarizes a case of IgAN diagnosed in a 15-year-old Hispanic male who presented to the ED with complaints of fever starting three days prior. The patient also expressed a sore throat, headache and most notably gross hematuria that commenced the night before. Noticeably, the patient was diagnosed with streptococcal pharyngitis a week prior. During their inpatient stay, the patient continued to have hematuria. A percutaneous kidney biopsy under real-time ultrasound guidance confirmed the diagnosis of IgAN. The pathology report consisted of mild mesangial hypercellularity due to diffuse 2+ granular mesangial staining noted with IgA with accompanying 2-3+ staining with C3 and lambda and trace 1+ staining with kappa. There were a few small intratubular cast stains for IgA and equally for kappa and lambda, confirming a diagnosis of IgAN. The pathogenesis of IgAN is hypothesized by four hit processes. IgAN has been noted to progress one’s risk of developing ESRD. Therefore, IgAN management is vital. This case report provides a great review of IgAN, aiding healthcare providers with more understanding of IgAN pathology and management.
